# Toxoplasmosis as a complication of transfusion in hemodialysis patients

**Published:** 2014-03-15

**Authors:** A Ebrahim Zadeh, T Bamedi, S Etemadi, M Shahrakipour, Kh Saryazdipour

**Affiliations:** 1 Department of Mycology and Parasitology, Tropical and Infectious Diseases Research Center,Zahedan University of Medical Sciences, Zahedan, Iran; 2 PhD Studentin Medical Parasitology, Department ofMedical Parasitology, School of Public Health, TehranUniversityof Medical Sciences,Tehran, Iran; 3 Department of biostatics and epidemiology, faculty of health, Zahedan University of Medical Sciences, Zahedan, Iran

**Keywords:** ELISA, Hemodialysis, Serology, Toxoplasmosis

## Abstract

**Background:**

Toxoplasma Gondi is an obligate intracellular protozoan parasite that is one of the most important protozoa of blood and tissue. The medical importance of this parasite is considered from two aspects of congenital defects and opportunities among those with congenital immune deficiency. Depending on the mode of transmission through blood and the risk of infection to Toxoplasma Gondi in hemodialysis patients, this serological study was conducted on Iranian population.

**Materials and Methods:**

This case-control study was conducted on 37 patients that underwent regular hemodialysis that 21 were male and 16 were female, and the mean age of them was 17.52±4.10 years (rages 13-22 years).

Thirty-seven healthy individuals were chosen as control group. All samples were tested by using of ELISA kits with two methods of IgG-ELISA and IgM-ELISA.

Finally obtained data was analysis by SPSS software.

**Results:**

The results of this study revealed high prevalence of toxoplasmosis among hemodialysis patients. Other findings indicate that 21 out of 37 patients in the case group were positive for anti-Toxoplasma Gondi IgG in case group while in control group just 11 individuals were positive that was a statistically significant difference(p <0.05 ).

**Conclusion:**

because of the high prevalence of toxoplasmosis among hemodialysis patients, identification of these patients for prevention of transfusion complications is important.

## Introduction

Toxoplasma Gondi is an obligate intracellular protozoan parasite which is one of the most important protozoa of blood and tissue. Sexual cycle of the parasite passes in main host (cats) that leads to the formation of oocytes, and its excretion via stool. Humans and other warm-blooded animals as intermediate host, indirectly infects by the parasite, and asexual cycle lead to cyst formation (1). This parasite typically through forms of the tachyzoite, tissue cysts and oocysts causes contamination. Orally, placental blood, leukocytes, transplantation and rarely needle stick in laboratory events were main causes of disease transmition (2-5). The prevalence of toxoplasma gondii varies according to the age, geographical location, moisture and patients' dietary habits. Approximately 1/3 of people are infected with this parasite worldwide. Results of serologic studies indicate that toxoplasmosis is the most common human infection in many parts of the world. Between different groups in different regions of the world population, the prevalence of infection is between 0 to 95 percent. In Iran the prevalence of toxoplasmosis is between 50 to 75 percent (6-7). Medically important of protozoan is considered from two aspects: 1- Congenital and 2 - being opportunistic in immune compromised people.

With this in mind, the clinical signs and symptoms depends on the host immune status. In people with healthy immune systems, the self-limited infection manifests itself. In people with impaired immune systems, particularly those with impaired cellular immune systems, there is the risk of infection recurrence or spread of infection (9-8).

Patients with malignancy, patients undergoing renal transplant, patients who treated with immunosuppressive drugs and hemodialysis patients with chronic renal failure are susceptible to Toxoplasma infection (10-13). Thus the identification of these individuals (patients) is important.

Overall, due to high prevalence of this parasite in Iran, and because of the involvement of the nervous system in immune compromised patients this disease is a crucial health point. But the physician may consider the diagnosis of toxoplasmosis away that causes importance health care and social problems. Thus the aim of this study was to determine IgM and IgG anti-Toxoplasma Gondi antibodies in hemodialysis patients in South-East of Iran (Zahedan).

## Materials and Methods

This case-control study was conducted during Murch to July 2013 on 37 patients that underwent regular hemodialysis in hemodialysis department. Thirty-seven healthy individuals without any history of renal failure were chosen as control group. All patients in this period of time were included in the study and patients who did not cooperate during the study were excluded from the study. The same numbers of healthy individuals were selected as control group in order to design a case-control study. Serum samples of both groups were stored at 4 ° C until the time of serological tests. Demographic and medical data including, sex, age, and medical history of both groups was recorded. After completion of sampling, all samples were simultaneously withdrawn of freezer and by using of ELISA kits (ADAlist Italia) with two methods of IgG-ELISA and IgM-ELISA were tested. 

The sensitivity and specificity of used IgM and IgG antibody assays were more than 98%. 

According to the kit instructions, in this method, specimens with serum level less than 10 Iu/ml IgG antibodies against Toxoplasma Gondi was considered negative and more than 10 lu/ml was considered positive. In the case of IgM antibodies against Toxoplasma Gondi serum level of less and more than 1lu/ml was considered negative and positive respectively. To verify the results, serums were stored at -70 until the end of work and suspected cases was tested again. The results of ELISA were read by Anthos 2020 labtec instrument Bustrial of Austria. 

**Statistical analysis**

After recording of data, all information and test results, using SPSS version 18 software and chi-square test was analyzed. PV ≤ 0.05 was considered statistically significant.

## Results

The results of this study indicated or showed that out of 37 hemodialysis patients, 21 were male and 16 were female, and the mean age of them was 17.52±4.10 years (rages 13-22 years). In the control group, 18 were male and 19 were female with the mean age of 20±5.21 years (ranges 15-25 years). Other findings of this study indicated that 21 out of 37 patients in the case group and 11 out of 37 patients in the control group were positive for anti-Toxoplasma Gondi IgG antibodies.

The results of this study indicate that the percentage of individuals who were positive for the presence of IgG antibodies in the group of patients, were significantly different than the control group (p <0.05). 

In addition, 5 hemodialysis patients were positive for anti-Toxoplasma Gondi IgM antibodies. In contrast, all subjects in the control group were negative, for the presence anti-Toxoplasma Gondi IgM antibodies. Significant differences between the two groups in term of anti-Toxoplasma Gondi IgM antibodies was observed (p <0.05) (Table 1)

**Tabel I:** Serologic Results test of anti-Toxoplasma Gondi IgM antibody in case and control groups

**p-value**	**Odd Ratio**	**Healthy individuals (%)**	**Hemodialysis** **patients (%)**	**anti-** **Toxoplasma Gondi ** **antibody**
**0.036**	4.5%; 95% CI 3.5±5.5	11 (29.7%)	21 (56.7%)	IgG antibody
**0.054**	1.2 1%; 95% CI.01±1.46	0 (0.0%)	5 (13.5%)	IgM antibody

## Discussion

Toxoplasma Gondi is most common protozoan that causes opportunistic infections in immune compromised patients and significant morbidity and mortality in these individuals Imposes substantial costs to the health care system. Since in the prevalence and incidence of kidney disease is on the rise, so the number of patients has reached from the 238 (9/49%) in 2000 to 375 cases (8/63%) in 2006. More than 24,000 people are diagnosed with this disease and their number is on the rise in recent years (14). It was shown that, the humoral and cellular immune systems in affected patients were suppressants and the number of circulating T cell was reduced. Hemodialysis cannot be returned to impair immune status (15-18). These factors are likely contributed to immunosuppression which causes high prevalence of latent infections and the high rate of mortality in these patients (21). This study revealed a high prevalence of Toxoplasma Gondi in hemodialysis patients and titer of antibodies confirmed this high prevalence. Titer of IgG anti-Toxoplasma Gondi antibodies in hemodialysis patients was statistically significant than in the control group. But in the control group IgM antibody was negative that comparison or comparing with control group was statistically significant.

Little research has been performed in the field. In the study of Solhjoo et al, 31 out of 44 patients undergoing Hemodialysis were found anti-Toxoplasma antibodies in their serum. According to the results, 26 patients had IgG-type antibodies against Toxoplasma, 3 patients had IgM, 3 patients had IgA antibody. In the control group of 44 patients, 16 patients had IgG-type antibodies against Toxoplasma Gondi, and IgM and, IgA Anti-Toxoplasma were negative (19). In the present study, the prevalence of IgG and IgM anti-Toxoplasma Gondi in hemodialysis patients and the control group was less than Solhjoo et al study. In the study of Abass et al, from 60 patients, Toxoplasma antibodies in 3/38% of patients and 15% of control group were 

seen which a significant difference was observed. In this study suggested that patients with chronic renal failure who were undergoing Hemodialysis to prevent Toxoplasma Gondi infection before Hemodialysis, patients being tested (20). The results of this study differ from the results that were observed by Abbas et al in 1996. In the study of Yazar et al in Turkey, from total of 100 hemodialysis patients, 97 patients(56%) had IgG antibodies against Toxoplasma Gondi, and (73/1%) of them had anti-Toxoplasma Gondi IgM (10). This study was showed higher prevalence of IgM antibodies in hemodialysis patients compared with Yazar study in 2003. In the study of Ocak et al, from 255 hemodialysis patients, 195 individuals had IgG and IgM anti-Toxoplasma antibodies (21). These results were different from the result of our study and revealed a higher prevalence of anti-Toxoplasma IgG and IgM antibodies.

## Conclusion

Hemodialysis patients should be regularly monitored to avoid the risk of acute Toxoplasmosis. 

## Conflict of interest

The authors have no conflict of interest.
